# Investigating continuation of folic acid supplementation during peri-conceptional period: a community-based cross-sectional study

**DOI:** 10.1186/s12978-023-01564-5

**Published:** 2023-02-20

**Authors:** Yanyan Mao, Hong Hu, Dongyan Chen, Yuhang Fang, Jun Liu, Min Li, Weijin Zhou

**Affiliations:** 1 NHC Key Laboratory of Reproduction Regulation, Shanghai Institute for Biomedical and Pharmaceutical Technologies, Shanghai, 200237 China; 2 Community Health Service Center of Jing-An-Si, Jing-an District, Shanghai, 200040 China; 3 Community Health Service Center of Pengpu Estate, Jing-an District, Shanghai, 200435 China; 4 NHC Key Laboratory of Birth Defects and Reproductive Health (Chongqing Population and Family Planning Science and Technology Research Institute), Chongqing, 400020 China

**Keywords:** Folic acid, Supplementation, Continuation, Preconception, Healthcare

## Abstract

**Background:**

Maternal folate may not reach an optimal level to prevent neural tube defects if supplementation commenced post-conception or took place pre-conception only. Our study aimed to investigate the continuation of folic acid (FA) supplementation from pre-conception to post-conception during peri-conceptional period and to examine its differences in FA supplementation between the subgroups taking the initiation timing into consideration.

**Methods:**

This study was conducted in two community health service centers in Jing-an District of Shanghai. Women accompanying their children to pediatric health clinics of the centers were recruited and asked to recall information concerning their socioeconomic and previous obstetric characteristics, utilization of healthcare and FA supplementation before and/or during pregnancy. The continuation of FA supplementation during peri-conceptional period were categorized into three subgroups: Supplementing with FA pre- and post-conception; supplementing with FA preconception only or post-conception only; no FA supplements pre-conception and post-conception. The relationship between FA continuation and couples’ characteristics were examined as setting the first subgroup as the base reference.

**Results:**

Three hundred and ninety-six women were recruited. Over 40% of the women started FA supplementation after conception and 30.3% of them supplemented with FA from pre-conception to the first trimester of their pregnancy. Compared to this one-third of participants, women who didn’t supplemented with any FA during peri-conceptional period were more likely to have no utilization of pre-conception healthcare ($OR$= 2.47, 95% $CI$: 1.33–4.61) or antenatal care ($OR$= 4.05, 95% $CI$: 1.76–9.34), or who had a lower family socioeconomic status ($OR$= 4.36, 95% $CI$: 1.79–10.64). Women who supplemented with FA pre-conception only or post-conception only were more likely to have no utilization of pre-conception healthcare ($OR$= 2.94, 95% $CI$: 1.79–4.82), or to have no previous pregnancy complication ($OR$=1.80, 95% $CI$: 0.99–3.28).

**Conclusion:**

Over two-fifth of the women started FA supplementation and only one-third of them had an optimal supplementation from pre-conception to the first trimester. Maternal utilization of healthcare before or during pregnancy together with maternal and paternal socioeconomic status may play a role in the continuation to FA supplementation pre- and post-conception.

**Supplementary Information:**

The online version contains supplementary material available at 10.1186/s12978-023-01564-5.

## Introduction

Folic acid (FA) supplementation plays an important role in reproductive health on a global scale [1], and has been associated with a dramatic reduction in risk of neural tube defects (NTDs) [2] and other adverse pregnancy outcomes [3]. Internationally, many governments recommend women take ≥ 400 μg of folic acid daily during the peri-conceptional period (from at least 4 weeks before conception until 12 weeks thereafter) according to the guideline by World Health Organization (WHO) [4, 5]. The prevalence of pre-conception folic acid supplementation is highly variable worldwide (North America: 32–51%; Europe: 9–78%; Asia: 21–46%; Middle East: 4–34%; Australia/New Zealand: 32–39%; Africa: 0%) and is relatively lower compared to during pregnancy [6]. Women from developing countries are less likely to have had pre-conception FA supplementation when compared to their counterparts from developed countries [7]. They may be less likely to adhere to FA supplementation after conception, which may lead to challenges in reaching sufficient folate level for NTDs prevention. Folate level of women who started the supplementation before pregnancy would more likely to reach an optimal level to prevent NTDs due to their high adherence to FA supplementation after pregnancy. In contrast, the level of the women who had a supplementation commenced after confirmation of pregnancy would less likely to have an optimal level and would miss an optimal window for NTDs prevention because neural tube closure may occur before many women are aware of their pregnancy [8].

FA supplementation before or during early pregnancy has been associated with a series of factors. [9–16]. Timely utilization of healthcare for pregnancy is likely to predict earlier and/or higher FA supplementation [12, 14–16]. Maternal peri-conceptional supplementation with FA has also been related to the spouse’s characteristics [9, 17]. High paternal socioeconomic status (SES) was associated with increased prevalence of maternal peri-conceptional FA supplementation. However, limited studies considered the starting time of FA supplementation when the studies investigated its continuation from pre-conception to post-conception during peri-conceptional period [9, 11], and few examined the association between utilization of preconception healthcare and the continuation of FA supplementation [18].

The present study, thereafter, investigated maternal continuation of FA supplementation during peri-conception period and identified its subgroups according to the initiation timing to examine their association with couples' characteristics especially the utilization of preconception healthcare and family SES.

## Materials and methods

### Participants

We used two method to calculated the sample size in this cross-sectional study. The necessary sample size was calculated using Raosoft [19] with the ideal sample size estimated to be 348 participants, based on a population size of 47,900 births from 2012 to 2017 in Jing-an District, 5% margin of error, 95% confidence level, and 35% prevalence of pre-conception FA supplementation. The sample size calculated by Design efficiency and prevalence of FA supplementation was 187 participants for each center and 374 ones in total (Additional file 1: Appendix S1).

This study was conducted from March to September 2018 in two community health service centers. The two centers were randomly selected from totally 14 centers in Jing-an District of Shanghai. All of the women with their children less than 6 years old were invited to participate the study when they visited the health center for a routine physical examination or vaccination according to the national infant and child healthcare scheme. The women and their children were interviewed after they agreed on and signed the consent form. Four hundred and three mother–child pairs finished the interview. After excluding women with missing data on FA supplementation, age, education, household registration, body mass index (BMI) and healthcare before or during pregnancy (n = 7), a total of 396 mother–child pairs were included in the final analysis.

This study was approved by the Institutional Review Board of Shanghai Institute of Planned Parenthood Research (PJ2014-33) on April 11th, 2014. Written informed consent was obtained from all subjects.

### Data collection for maternal FA supplementation before or during pregnancy

Seven to eight paediatricians in each of the two centers were employed to recruit and interview the participants. All the employed paediatricians were trained on a specific seminar before recruitment. The participants were interviewed face to face to complete a structured questionnaire Participants were asked about FA supplementation through the following four questions:

1. Did you supplement with FA prior to conception?
2. When did you start to supplement with FA? (one/three/six months prior to conception)
3. Did you supplement with FA during pregnancy?
4. When did you start to supplement with FA? (the first trimester/second/third trimester)

According to the four questions, women was categorized into three subgroups: 1) Women who supplemented with FA prior to conception and in the first trimester of pregnancy was defined as ‘Optimal supplementation’ group; 2) women who had the supplementation only prior to conception or only in the first trimester as ‘Suboptimal supplementation’ group; 3) Women who had no supplementation during peri-conceptional period as ‘No supplementation’ group. The optimal group had a better continuation of FA supplementation and would have an earlier initiation time, a longer duration of supplementation or a higher accumulative dose of FA intake than the sub-optimal group or no supplementation group.

### Data collection for utilization of healthcare before or during pregnancy

Two questions were designed to investigate utilization of pre-conception healthcare and antenatal care:

1. Did you have a pre-conception health examination for this pregnancy within 6 months prior to the conception? (Utilization of pre-conception healthcare)
2. Did you have antenatal care during pregnancy? (Utilization of antenatal care)

The participants reported the birth-date, places of household registration, education level, family income at the time of delivery of themselves and their husbands. They also reported their previous pregnancy complication, BMI before pregnancy and their child’s date of birth in the interview. Pregnancy complication included spontaneous abortion, induced abortion, or still birth / still born.

### Statistical analysis

The outcome variable was FA supplementation prior to conception or in the first trimester, which was grouped into three categories (no supplementation, supplementation only prior to conception or only in the first trimester, and supplementation from preconception till the first trimester). The potential factors associated with FA supplementation as follows: Utilization of pre-conception healthcare and antenatal care was grouped into two categories (yes and no); Parental age at childbirth was calculated using the mother and their child’s date of birth, respectively and grouped into two categories (< 30 and ≥ 30 years); Parental SES was measured by places of household registration (local or non-local), education level (high school or below, college, or university and above); Paternal monthly income (≤ 1 and > 1 × 10 000 ¥) and annual family income (< 20 and ≥ 20 × 10 000 ¥) were both grouped into two categories; Maternal obstetrical characteristics included maternal body mass index (BMI) before pregnancy (< 18.5, 18.5–23.9, > 23.9 $kg/{m}^{2}$), and gravidity at the pregnancy (0 and 1 +). Child’s age was also grouped into three categories (0–1, 2–3, and 4 + years).

For parental SES, the variables considered in the study were correlated to a certain extend, therefore, a factor analysis was applied to identify the potential SES variables (factors) based on these known variables. Then an integrated score of SES for each participant was calculated according to the score and weight of each identified factor. The integrated score of SES we estimated was negatively correlated with the SES. Then participants were categorized into four groups in terms of the quantile of the SES score. All results of factor analysis shown in Additional file 1: Appendix S1.

The association between FA supplementation and each of the above-mentioned factor was evaluated using chi-square test. The factors with a statistical significance ($P$≤ 0.05) were further estimated together in a multivariate multinomial logistic regression model. This analysis was to differentiate optimal supplementation group from sub-optimal or no supplementation group. Further, nineteen pediatricians were finally employed to interview participants and the information may also be biased by various interviewers although they were trained before interviewing the mothers. Therefore, a sensitivity analysis was applied by using the cluster option in Stata. The cluster method took correlation of mothers interviewed by the same pediatrician into consideration for estimating standard error.

Odds ratios ($OR$ s) and their 95% confidence intervals ($CI$ s) were estimated, z-statistics were used to evaluate the differences in the $OR$ s and 95% $CI$ s, and values of $P$≤ 0.05 were considered to be statistically significant. All statistical analysis was performed using Stata 15.1 software (Stata Corp, Texas, USA).

## Results

### Maternal and children characteristics

The description of parental and children’s characteristics was listed in Table 1. Of the 396 participants, 40.15% utilized pre-conception healthcare and 86.11% utilized antenatal care. More than half of the women were younger than 30 years old (57.32%), were local residents (61.87%), had a bachelor’s degree or higher (89.65%), and had an annual household income of less than 200, 000 Chinese Yuan (59.60%). The majority of the women had given birth within the last 4 years (76.03%),70.71% of them had normal BMI, nearly 70% were primipara and nearly 20% had previously experienced pregnancy complication. Their spouses had the similar demographic characteristic distribution except for the age. More than 55% of the spouses were older than 30 years age and nearly 50% of them earned less than 10, 000 Chinese Yuan. According to the integrated score of family SES, the women in G1 and G2 accounted for about 60% (25% and 35.61%, respectively) of the 396 women.

**Table 1:** Parental and children’s characteristics (n = 396)

Characteristics	N	%
Maternal age (years)		
< 30	227	57.32
≥ 30	169	42.68
Maternal household registration		
Local	245	61.87
Non-local	151	38.13
Maternal education		
High school or below	41	10.35
College or above	355	89.65
Gravidity		
0	274	69.19
1 ~	122	30.81
Maternal BMI before pregnancy ($kg/{m}^{2}$)		
< 18.5	76	19.19
18.5–23.9	280	70.71
> 23.9	40	10.10
Previous pregnancy complication^a^		
Yes	74	18.69
No	322	81.31
Utilization of pre-conception health care		
Yes	159	40.15
No	237	59.85
Utilization of antenatal care		
Yes	341	86.11
No	55	13.89
Paternal age (years)		
< 30	177	44.70
≥ 30	219	55.30
Paternal household registration		
Local	271	68.43
Non-local	125	31.57
Paternal education		
High school or below	35	8.84
College or above	361	91.16
Paternal monthly income (× 10 000 ¥^b^)		
≤ 1	198	50.00
> 1	198	50.00
Annual household income (× 10 000 ¥^b^)		
< 20	236	59.60
≥ 20	160	40.40
Family SES scores and groups		
− 0.4478 ~ (G1)	99	25.00
− 0.1297~ (G2)	141	35.61
0.3184 ~ (G3)	57	14.39
0.3184 ~ (G4)	99	25.00
Age of child (years)		
0–1	152	38.38
2–3	149	37.63
4 ~	95	23.99
Folic acid supplementation during periconception period	308	77.78
Supplementation prior to conception and in the first trimester	120	30.30
Supplementation only prior to conception	22	5.56
Supplementation only in the first trimester	166	41.92

^a^Previous pregnancy complication includes stillbirth and spontaneous or induced abortion

^b^ ¥, Chinese Yuan

### Folic acid supplementation and potential risk factors

Of the 396 participants, 77.78% (308/396) of the women who participated in the study supplemented with FA prior to conception or in the first trimester and 84.5% (120/142) of the women who supplemented with FA prior to conception adhered to FA during pregnancy. However, approximately 35% started before pregnancy, 30.3% of the women had supplementation prior to conception and in the first trimester, and more than 40% of the women supplemented with FA started after conception (Table 1 and Additional file 2: Appendix S2).

Chi-square test of the association between FA supplementation and each potential factor was shown in Table 2. Results indicated that FA supplementation during peri-conceptional period was significant associated with maternal age, household registration, education, and annual family income. Women who were older, were local residents, had a higher education or had a higher family income, were more likely to supplement with FA from preconception till the first trimester. The results also suggested that a significant association between maternal utilization of health care before or during pregnancy, previous pregnancy complication and FA supplementation. Women who utilized health care before or during pregnancy or experienced any pregnancy complication, had a higher probability of FA supplementation prior to conception and in the first three month of pregnancy. Women whose husbands were older, were local residents and had a high monthly income, were also had a higher probability of FA supplementation prior to conception and in the first three month of pregnancy.

**Table 2:** Association between peri-conceptional folic acid supplementation and characteristics of children and their parents (n = 396)

	FA supplementation during peri-conception period						${\chi }^{2}$	$P$
	No supplementation		Sub-optimal supplementation		Optimal supplementation			
	(n = 88)		(n = 188)		(n = 120)			
	n	%	n	%	n	%		
Maternal age (years)							13.03	0.001
< 30	59	67.05	115	61.17	53	44.17		
≥ 30	29	32.95	73	38.83	67	55.83		
Maternal household registration							12.92	0.002
Local	40	45.45	125	66.49	80	66.67		
Non-local	48	54.55	63	33.51	40	33.33		
Maternal education							6.90	0.032
High school or below	15	17.05	19	10.11	7	5.83		
College or above	73	82.95	169	89.89	113	94.17		
Gravidity							2.83	0.243
0	64	72.73	134	71.28	76	63.33		
1 ~	24	27.27	54	28.72	44	36.67		
Maternal BMI before pregnancy ($kg/{m}^{2}$)							5.79	0.216
< 18.5	19	21.59	30	15.96	27	22.50		
18.5–23.9	56	63.64	142	75.53	82	68.33		
> 23.9	13	14.77	16	8.51	11	9.17		
Previous pregnancy complication^a^							6.108	0.047
No	76	86.36	157	83.51	89	74.17		
Yes	12	13.64	31	16.49	31	25.83		
Utilization of pre-conception health care							21.93	< 0.001
No	57	64.77	129	68.62	51	42.50		
Yes	31	35.23	59	31.38	69	57.50		
Utilization of antenatal care							26.77	< 0.001
No	27	30.68	18	9.57	10	8.33		
Yes	61	69.32	170	90.43	110	91.67		
Paternal age (years)							7.43	0.024
< 30	49	55.68	84	44.68	44	36.67		
≥ 30	39	44.32	104	55.32	76	63.33		
Paternal household registration							11.86	0.003
Local	47	53.41	136	72.34	88	73.33		
Non-local	41	46.59	52	27.66	32	26.67		
Paternal education							5.36	0.068
High school or below	13	14.77	15	7.98	7	5.83		
College or above	75	85.23	173	92.02	113	94.17		
Paternal monthly income (× 10 000 ¥^b^)							9.43	0.009
≤ 1	54	61.36	96	51.06	48	40.00		
> 1	34	38.64	92	48.94	72	60.00		
Annual household income (× 10 000 ¥^b^)							6.87	0.032
< 20	63	71.59	107	56.91	66	55.00		
≥ 20	25	28.41	81	43.09	54	45.00		
Family SES							23.48	0.001
G1	12	13.64	54	28.72	33	27.50		
G2	25	28.41	67	35.64	49	40.83		
G3	13	14.77	27	14.36	17	14.17		
G4	38	43.18	40	21.28	21	17.50		

^a^Previous pregnancy complication includes stillbirth and spontaneous or induced abortion

^b^ ¥, Chinese Yuan

### Multivariate regression analysis for differentiating sub-groups of folic acid supplementation

Multinomial regression analysis for differentiating subgroups of FA supplementation prior to conception and /or in the first trimester of pregnancy was presented in Table 3. Compared to women in ‘optimal supplementation’ group, women in no FA supplementation group were less likely to utilize pre-conception healthcare ($OR$=2.47, 95%$CI$:1.33–4.61) or antenatal care ($OR$=4.05, 95%$CI$:1.76–9.34). They were more likely to be in the family SES of G4 ($OR$=4.36, 95%$CI$=1.79–10.64). Women in ‘sub-optimal supplementation’ group were also less likely to utilize pre-conception healthcare ($OR$=2.94, 95%$CI$:1.79–4.82), to be younger than 30 ($OR$=1.82, 95%$CI$:0.95–3.48) and to have suffered from pregnancy complication previously ($OR$=1.80, 95%$CI$:0.99–3.28) compared to the reference group.

**Table 3:** Multinomial logistic regression differentiating subgroups of folic acid (FA) supplementation prior to conception and/or in the first trimester of pregnancy (based on ‘Optimal supplementation’)

	No supplementation						Sub-optimal supplementation					
	$B$^a^	$SE$^a^	$Z$	$OR$^a^	95% $CI$^a^	$P$	$B$^a^	$SE$^a^	$Z$	$OR$ ^a^	95% $CI$^a^	$P$
Maternal age (years)												
< 30 vs. ≥ 30	0.61	0.40	1.51	1.83	0.83–4.02	0.132	0.60	0.33	1.82	1.82	0.95–3.48	0.069
Paternal age (years)												
< 30 vs. ≥ 30	0.11	0.39	0.27	1.11	0.52–2.39	0.789	− 0.23	0.33	− 0.70	0.79	0.41–1.52	0.483
Family SES												
G2 vs. G1	0.15	0.44	0.33	1.16	0.49–2.73	0.740	− 0.34	0.31	− 1.12	0.71	0.39–1.30	0.265
G3 vs. G1	0.39	0.53	0.73	1.48	0.52–4.21	0.465	− 0.13	0.42	− 0.33	0.88	0.40–1.93	0.743
G4 vs. G1	1.47	0.46	3.44	4.36	1.79–10.64	0.001	0.11	0.37	0.30	1.12	0.54–2.29	0.766
Previous pregnancy complication												
No vs. Yes	0.71	0.41	1.73	2.03	0.91–4.52	0.084	0.59	0.30	1.94	1.80	0.99–3.28	0.053
Utilization of pre-conception health care												
No vs. Yes	0.90	0.32	2.85	2.47	1.33–4.61	0.004	1.07	0.25	4.27	2.94	1.79–4.82	< 0.001
Utilization of antenatal care												
No vs Yes	1.40	0.43	3.28	4.05	1.76–9.34	0.001	0.00	0.43	1.00	1.80	0.43–2.32	1.000

^a^$B$ coefficient; $SE$ standard error; $OR$ odd ratio; $CI$ Confidence interval

The other parental characteristics were not associated with the two sub-groups of FA supplementation after multivariate analysis although younger age of women or their previous pregnancy complication may be associated with sub-optimal and no FA supplementation respectively. Majority of the results in multivariate analysis retained in the sensitivity analysis which set the family SES characteristics independently (Additional file 3: Appendix S3, Tables S3-1).

## Discussion

The present study highlighted that over 20% of the women who did not supplement with FA at all, over 40% starting after conception and only 30% supplemented with FA according to WHO recommendation (before conception to the end of the first trimester) [4], although majority of the women who started pre-conception continuously had an FA supplementation after conception. The prevalence of FA supplementation meeting the recommendation (Optimal supplementation) in this study was lower than the one among the rural women from Chinese National Free Pre-conception Health Examination Project (NFPHEP) (41.92% vs 52.73%) while the prevalence of the supplementation starting after conception was higher (41.92% vs 22.13%) [20]. The prevalence of the supplementation starting before conception in our study was seemingly lower than the one among the women from 14 counties of Shanghai in 2016 (35.9% vs 42.8%) [21], unfortunately who did not report the continuation of FA supplementation during peri-conceptional study.

In further analysis, under-utilization of preconception healthcare was not only associated with no supplementation but also associated with suboptimal supplementation compared to optimal supplementation which was defined as supplementation with FA prior to conception and in the first trimester of pregnancy. Previous studies in different countries have found that utilization of pre-conception healthcare has been a predictor for higher supplementation of FA prior to conception [14–16, 22], but None of the studies analyzed its association with the continuation of FA supplementation from pre-conception to post-conception. Liu, et al. observed that in Northwest of China women who had a participation of health counselling that related to maternal healthcare and fetal development before or during pregnancy would be more likely to supplement with FA continuously longer than 180 days during peri-conceptional period but they did not dissect the initiation timing of FA supplementation [23]. In the present study, the subgroups of FA supplementation during peri-conceptional period was identified according to initiation timing and continuation of FA supplementation.

Preconception healthcare evaluates a woman’s (or the couple’s) risk before pregnancy, in order to reduce the risk for adverse maternal and infant outcomes [24]. Folic acid counselling is one of its essential components, which plays an important role in initiation of FA supplementation prior to conception, and may thereafter bring out higher adherence to FA during the pregnancy. Chinese NFPHEP was put in place nationwide since 2010 and it has been to provide free health examinations and counselling prior to conception for childbearing couples who are planning to conceive [25]. The present study based on Jing-an District in Shanghai’s downtown, which was the pilot of Chinese NFPHEP, provided the preconception health services since 2011 and thereafter all of the counties in Shanghai provided the services since 2013. Maternal awareness of folic acid has been identified as a strong predictor of pre-conception folic acid use [24]. Provision and utilization of pre-conception healthcare may enhance women’s FA knowledge [26, 27] and promote women to start FA supplementation earlier than they originally would have [20]. Women started supplementing with FA earlier might have a good compliance to FA supplements. In our study, most of the women (84.5%) who supplemented with FA prior to conception adhered to FA during pregnancy. High utilization of pre-conception healthcare was also associated with a higher utilization of antenatal care [14]. Late utilization of antenatal care and fewer antenatal visits have been associated with later and lower FA supplementation [11] and early registration for antenatal care has been associated with higher iron-folate taking [28]. Higher frequency of antenatal visits has been related to a longer duration of micronutrient use before and during pregnancy [23].

In addition, we combined parental SES characteristics into an integrated index and found that lower family SES was associated with higher risk of no FA supplementation than the optimal group. Higher educational levels, higher family income levels and urban residents were associated with high duration or dosage of micronutrient supplementation or FA supplementation [10, 18, 23]. The SES variable of these previous studies were analyzed independently while we considered their correlation. Moreover, advanced age, previous pregnant intention or obstetric experiences may also be related to the duration of FA supplementation [13, 29]. Our findings were seemingly consistent with them but some of the results were not statistically significant.

## Strengths and limitations

As of now, the present study is the first of its kind to evaluate the association between utilization of preconception healthcare and the adherence to FA supplements during peri-conceptional period as taking the initiation timing of FA supplementation among Chinese women. However, the following limitations should be considered when the results were elucidated. Firstly, the existing sample size limited the power to examine more subgroups according to initiation timing of FA supplementation because we did not take the prevalence of each parental characteristics into consideration when estimating the sample size. Secondly, there was no more information on frequency of preconception counselling or antenatal visit or on maternal FA supplementation to estimate their dose–response relationship and also was no more information on other known confounders. Thirdly, most of the women participating in this study gave the birth beyond one year (61.77%). The information they provided may be biased by recall [30], but no relationship was observed between FA supplementation and child age [31]. The estimate may also be biased by the multi-interviewers of 19 although they were well-trained. We controlled the correlation of the same interviewer using the cluster option and most of the findings persisted in the sensitivity analyses (Additional file 3: Appendix S3, Table S3-2).

## Conclusions

The adherence to the supplementation post-conception for women who supplemented with folic acid starting pre-conception was relatively high but over two-fifth started after conception and only one-third of the women had an optimal supplementation. The findings of identifying the characteristics of the women who had no or suboptimal FA supplementation suggested that maternal utilization of healthcare before or during pregnancy together with previous pregnancy complication and family SES may play a role in FA supplementation during peri-conceptional period, and maternal utilization of pre-conception healthcare may be given special importance to for improve the initiation timing and the continuation of FA supplementation pre- and post- conception.

## Supplementary Information

**Additional file 1. **Calculating the sample size. Factor analysis.

**Additional file 2.** **Figure S2-1.** Flowchart of participants’ supplementation with FA prior to conception or in the first trimester.

**Additional file 3. Table S3-1.** Multinomial logistic regression differentiating subgroups of folic acid (FA) supplementation prior to conception and/or in the first trimester of pregnancy (based on ‘Optimal supplementation’). **Table S3-2. **Multinomial logistic regression differentiating subgroups of folic acid (FA) supplementation prior to conception and/or in the first trimester of pregnancy (based on ‘Optimal supplementation’).

## Data Availability

The datasets used and/or analyzed during the current study are available from the corresponding author on reasonable request.

## Abbreviations

FA: Folic acid
OR: Odds ratio
CI: Confidence interval
NTDs: Neural tube defects
WHO: World Health Organization
NFPHEP: National Free Pre-conception Health Examination Project
