# Supplementary material for: Investigating continuation of folic acid supplementation during peri-conceptional period: a community-based cross-sectional study
Source: Reprod Health. 2023 Feb 20;20:34. doi: 10.1186/s12978-023-01564-5 (PMC9942345; doi:10.1186/s12978-023-01564-5)
Supplement: Supplementary file 1 — Additional file 1. Calculating the sample size. Factor analysis. [file 12978_2023_1564_MOESM1_ESM.docx]

**Additional file 1**

**1、****Calculating the sample size**

The sample size of the cross-sectional study can also be estimated on the following parameters:

1. The incidence of FA supplementation in the whole population of Shanghai or Jin’an ‘p’. If ‘p’ is unknown, setting p of 50% and a maximum sample size can be calculated. We did not know the incidence, thus we used 50%.
2. The level of significance ‘α’ which is the expected to reach. In this study, α=0.05.
3. The expected precision ‘d’, In this study, ‘d’ was set as 20% of ‘p’.
4. Design efficiency ‘D’, which was set as 2 in this study. The required sample size was calculated by the formula: n=D*p*(1-p)* (Zα/d)^2^.

Therefore, n is estimated to be 186.2. So, each center investigate at least 186.2 to achieve the desired design efficiency.

**2、****Factor analysis**

**Step 1 Bartlett test of sphericity & Kaiser-Meyer-Olkin Measure**

| **Test** | **Statistics** |
| --- | --- |
| **Determinant of the correlation matrix** |  |
|  | Det=0.228 |
|  |  |
| **Bartlett test of sphericity:** H0: variables are not intercorrelated | |
|  | Chi-square=579.181 |
|  | Degrees of freedom =15 |
|  | p-value=0.000 |
| **Kaiser-Meyer-Olkin Measure of Sampling Adequacy** |  |
|  | KMO=0.625 |

**Step 2 Factor analysis/correlation**

**Table S1-1 Initial statistics from principal components extraction**

| Factor | Eigenvalue | Difference | Proportion | Cumulative |
| --- | --- | --- | --- | --- |
| Factor1 | 2.3220 | 0.8165 | 0.3870 | 0.3870 |
| Factor2 | 1.5055 | 0.6062 | 0.2509 | 0.6379 |
| Factor3 | 0.8993 | 0.4023 | 0.1499 | 0.7878 |
| Factor4 | 0.4971 | 0.0423 | 0.0828 | 0.8706 |
| Factor5 | 0.4548 | 0.1335 | 0.0758 | 0.9464 |
| Factor6 | 0.3213 | . | 0.0536 | 1.0000 |

**Table S1-2 Factor loadings (pattern matrix) and unique variances before rotation**

| Variable | Factor1 | Factor2 | Uniqueness |
| --- | --- | --- | --- |
| Maternal education | 0.7668 | 0.0527 | 0.4093 |
| Paternal education | 0.6692 | 0.1947 | 0.5143 |
| Paternal monthly income | 0.3431 | 0.7609 | 0.3034 |
| Annual household income | 0.5019 | 0.5687 | 0.4246 |
| Maternal household registration | 0.6967 | -0.5194 | 0.2449 |
| Paternal household registration | 0.6568 | -0.541 | 0.2759 |

**Table S1-3 Rotated factor loadings (pattern matrix) and unique variances**

| Variable | Factor1 | Factor2 | Uniqueness |
| --- | --- | --- | --- |
| Maternal education | **0.5913** | **0.4911** | 0.4093 |
| Paternal education | **0.429** | **0.5492** | 0.5143 |
| Paternal monthly income | -0.1666 | **0.8179** | 0.3034 |
| Annual household income | 0.0746 | **0.7549** | 0.4246 |
| Maternal household registration | **0.8688** | -0.014 | 0.2449 |
| Paternal household registration | **0.8491** | -0.0549 | 0.2759 |

**Table S1-4 The statistics from principal components extraction after rotation**

| Factor | Variance | Difference | Proportion | Cumulative |
| --- | --- | --- | --- | --- |
| Factor1 | 2.0429 | 0.2583 | 0.3405 | 0.3405 |
| Factor2 | 1.7846 | . | 0.2974 | 0.6379 |

**Step 3** **Factor 1, 2 and the Integrated score**

**Table S1-5 Mean and quantiles of factor 1, 2 and the Integrated score**

| Variable | Obs | Mean | 5% | 25% | 50% | 75% | 95% | Min | Max |
| --- | --- | --- | --- | --- | --- | --- | --- | --- | --- |
|  |  |  |  |  |  |  |  |  |  |
| Factor 1 | 396 | 1.72E-09 | -0.9932 | -0.9005 | -0.5497 | 0.8666 | 2.0286 | -0.9932 | 2.5852 |
| Factor 2 | 396 | -1.63E-09 | -1.4994 | -1.0135 | -0.0220 | 0.8590 | 1.8209 | -1.4994 | 2.3322 |
| Integrated score | 396 | 5.88E-09 | -0.7659 | -0.4478 | -0.1297 | 0.3184 | 1.7758 | -0.7659 | 2.1536 |

**Step 4 SES integrated score and SES characteristics**

**Table S1-6 Mean and median of integrated score of family SES according to SES characteristics**

| **SES characteristics** | **SES integrated score** | | **Frequency** |
| --- | --- | --- | --- |
|  | **Mean** | **Median** |  |
| Family SES status |  |  |  |
| G1 | -0.6937 | -0.7659 | 99 |
| G2 | -0.2329 | -0.1297 | 141 |
| G3 | 0.0619 | 0.0003 | 57 |
| G4 | 0.9897 | 0.6366 | 99 |
| Maternal education |  |  |  |
| College or above | -0.1848 | -0.1297 | 355 |
| High school or below | 1.6003 | 1.7758 | 41 |
| Paternal education |  |  |  |
| College or above | -0.1508 | -0.1297 | 361 |
| High school or below | 1.5558 | 1.8786 | 35 |
| Maternal household registration |  |  |  |
| Local | -0.3585 | -0.4047 | 245 |
| Non-local | 0.5817 | 0.3616 | 151 |
| Paternal household registration |  |  |  |
| Local | -0.2901 | -0.3775 | 271 |
| Non-local | 0.6289 | 0.3616 | 125 |
| Paternal monthly income (× 10 000 ¥ ^b^) |  |  |  |
| >1 | -0.29203 | -0.4047 | 198 |
| ≤1 | 0.29203 | -0.1297 | 198 |
| Annual household income (× 10 000 ¥ ^b^) |  |  |  |
| ≥20 | -0.4752 | -0.4909 | 160 |
| <20 | 0.3222 | -0.0216 | 236 |
