# Supplementary material for: Investigating continuation of folic acid supplementation during peri-conceptional period: a community-based cross-sectional study
Source: Reprod Health. 2023 Feb 20;20:34. doi: 10.1186/s12978-023-01564-5 (PMC9942345; doi:10.1186/s12978-023-01564-5)
Supplement: Supplementary file 2 — Additional file 2. Figure S2-1. Flowchart of participants’ supplementation with FA prior to conception or in the first trimester. [file 12978_2023_1564_MOESM2_ESM.docx]

**Additional file 2: Appendix S2**

**Participants**

**（n=396）**

**Post-conception**

**(n=166)**

**No FA supplementation**

**（n=88）**

**FA supplementation during peri-conceptional period**

**（n=308）**

**Pre-conception only**

**（n=22）**

**Pre- & post-conception**

**（n=120）**

**Figure S2-1 Flowchart of participants’ supplementation with FA prior to conception or in the first trimester**
