# Supplementary material for: Investigating continuation of folic acid supplementation during peri-conceptional period: a community-based cross-sectional study
Source: Reprod Health. 2023 Feb 20;20:34. doi: 10.1186/s12978-023-01564-5 (PMC9942345; doi:10.1186/s12978-023-01564-5)
Supplement: Supplementary file 3 — Additional file 3. Table S3-1. Multinomial logistic regression differentiating subgroups of folic acid (FA) supplementation prior to conception and/or in the first trimester of pregnancy (based on ‘Optimal supplementation’). Table S3-2. Multinomial logistic regression differentiating subgroups of folic acid (FA) supplementation prior to conception and/or in the first trimester of pregnancy (based on ‘Optimal supplementation’). [file 12978_2023_1564_MOESM3_ESM.docx]

**Additional file 3: Appendix S3**

**Table S3-1 Multinomial logistic regression** **differentiating subgroups of folic acid (FA) supplementation prior to conception and/or in the first trimester of pregnancy (based on ‘Optimal supplementation’)**

|  | **No supplementation** | | | | | |  |  | **Suboptimal supplementation** | | | | |
| --- | --- | --- | --- | --- | --- | --- | --- | --- | --- | --- | --- | --- | --- |
|  | $\boldsymbol{B}$ **^a^** | $\boldsymbol{SE}$ **^a^** | $\boldsymbol{Z}$ | $\boldsymbol{OR}$ **^a^** | **95%**$\boldsymbol{CI}$ **^a^** | $\boldsymbol{P}$ |  | $\boldsymbol{B}$ **^a^** | $\boldsymbol{SE}$ **^a^** | $\boldsymbol{Z}$ | $\boldsymbol{OR}$ **^a^** | **95%**$\boldsymbol{CI}$ **^a^** | $\boldsymbol{P}$ |
| Maternal age (years) |  |  |  |  |  |  |  |  |  |  |  |  |  |
| <30 vs ≥30 | 0.60 | 0.40 | 1.48 | 1.82 | 0.82 -4.03 | 0.138 |  | 0.61 | 0.33 | 1.83 | 1.83 | 0.96 -3.50 | **0.067** |
| Maternal household registration |  |  |  |  |  |  |  |  |  |  |  |  |  |
| Non-local vs Local | 0.34 | 0.43 | 0.79 | 1.41 | 0.61 - 3.271 | 0.428 |  | -0.12 | 0.35 | -0.34 | 0.89 | 0.45 - 1.77 | 0.736 |
| Maternal education |  |  |  |  |  |  |  |  |  |  |  |  |  |
| High school or below vs College or above | 0.58 | 0.56 | 1.03 | 1.78 | 0.59 – 5.36 | 0.305 |  | 0.58 | 0.52 | 1.12 | 1.79 | 0.65 -4.95 | 0.262 |
| Previous pregnancy complication |  |  |  |  |  |  |  |  |  |  |  |  |  |
| No vs Yes | 0.64 | 0.41 | 1.55 | 1.91 | 0.85- 4.29 | 0.120 |  | 0.55 | 0.31 | 1.78 | 1.73 | 0.95 -3.17 | **0.074** |
| Paternal age (years) |  |  |  |  |  |  |  |  |  |  |  |  |  |
| <30 vs ≥30 | 0.08 | 0.39 | 0.21 | 1.09 | 0.50 – 2.35 | 0.834 |  | -0.24 | 0.33 | -0.71 | 0.79 | 0.41 -1.52 | 0.479 |
| Paternal household registration |  |  |  |  |  |  |  |  |  |  |  |  |  |
| Non-local vs Local | 0.65 | 0.44 | 1.49 | 1.91 | 0.81 – 4.49 | 0.137 |  | 0.24 | 0.36 | 0.66 | 1.27 | 0.62 – 2.60 | 0.511 |
| Paternal monthly income (× 10 000 ¥) ^a^ |  |  |  |  |  |  |  |  |  |  |  |  |  |
| ≤1 vs ＞1 | 0.71 | 0.35 | 2.00 | 2.03 | 1.01 –4.06 | **0.045** |  | 0.35 | 0.29 | 1.23 | 1.42 | 0.81 -2.50 | 0.219 |
| Annual family income (× 10 000 ¥) |  |  |  |  |  |  |  |  |  |  |  |  |  |
| <20 vs ≥20 | 0.04 | 0.36 | 0.11 | 1.04 | 0.50 – 2.13 | 0.910 |  | -0.32 | 0.29 | -1.13 | 0.72 | 0.41-1.27 | 0.257 |
| Utilization of pre-conception health care |  |  |  |  |  |  |  |  |  |  |  |  |  |
| No vs Yes | 0.86 | 0.32 | 2.69 | 2.36 | 1.25 - 4.44 | **0.008** |  | 1.04 | 0.26 | 4.07 | 2.83 | 1.72 - 4.68 | **<0.001** |
| No vs Yes | 1.41 | 0.43 | 3.30 | 4.11 | 1.77 – 9.50 | **0.001** |  | 0.05 | 0.43 | 0.11 | 1.05 | 0.45 - 2.44 | 0.910 |

^a^$B$, coefficient; $SE$, standard error; $OR$, odd ratio; $CI$, Confidence interval

**Table S3-2 Multinomial logistic regression differentiating subgroups of folic acid (FA) supplementation prior to conception and/or in the first trimester of pregnancy (based on ‘Optimal supplementation’)**

|  | **No supplementation** | | | | | |  |  | **Suboptimal supplementation** | | | | |
| --- | --- | --- | --- | --- | --- | --- | --- | --- | --- | --- | --- | --- | --- |
|  | $\boldsymbol{B}$ **^a^** | $\boldsymbol{SE}$ **^a^** | $\boldsymbol{Z}$ | $\boldsymbol{OR}$ **^a^** | **95%**$\boldsymbol{CI}$ **^a^** | $\boldsymbol{P}$ |  | $\boldsymbol{B}$ **^a^** | $\boldsymbol{SE}$ **^a^** | $\boldsymbol{Z}$ | $\boldsymbol{OR}$ **^a^** | **95%**$\boldsymbol{CI}$ **^a^** | $\boldsymbol{P}$ |
| Maternal age (years) |  |  |  |  |  |  |  |  |  |  |  |  |  |
| <30 vs ≥30 | 0.61 | 0.40 | 1.52 | 1.83 | 0.84 - 4.00 | 0.129 |  | 0.60 | 0.51 | 1.17 | 1.82 | 0.67-1.98 | 0.272 |
| Paternal age (years) |  |  |  |  |  |  |  |  |  |  |  |  |  |
| <30 vs ≥30 | 0.11 | 0.39 | 0.27 | 1.11 | 0.52 - 2.40 | 0.786 |  | -0.23 | 0.38 | -0.61 | 0.79 | 0.38 -1.67 | 0.541 |
| Family SES status |  |  |  |  |  |  |  |  |  |  |  |  |  |
| G2 vs G1 | 0.15 | 0.43 | 0.34 | 1.16 | 0.50 -2.70 | 0.736 |  | -0.34 | 0.41 | -0.83 | 0.71 | 0.31 -1.60 | 0.407 |
| G3 vs G1 | 0.39 | 0.67 | 0.58 | 1.48 | 0.40 -5.47 | 0.560 |  | -0.13 | 0.34 | -0.39 | 0.88 | 0.45 -1.70 | 0.696 |
| G4 vs G1 | 1.47 | 0.41 | 3.63 | 4.36 | 2.00-9.65 | **<0.001** |  | 0.11 | 0.37 | 0.30 | 1.12 | 0.54 -2.29 | 0.766 |
| Previous pregnancy complication |  |  |  |  |  |  |  |  |  |  |  |  |  |
| No vs Yes | 0.71 | 0.39 | 1.81 | 2.03 | 0.94- 4.36 | **0.070** |  | 0.59 | 0.24 | 2.49 | 1.80 | 1.13 -2.87 | **0.013** |
| Utilization of pre-conception health care |  |  |  |  |  |  |  |  |  |  |  |  |  |
| No vs Yes | 0.90 | 0.30 | 3.00 | 2.47 | 1.37- 4.47 | **0.003** |  | 1.07 | 0.43 | 2.49 | 2.94 | 1.26 - 6.86 | **0.013** |
| Utilization of antenatal care |  |  |  |  |  |  |  |  |  |  |  |  |  |
| No vs Yes | 1.40 | 0.63 | 2.23 | 4.05 | 1.18 – 13.88 | **0.026** |  | 0.00 | 0.58 | 0.00 | 1.80 | 0.32- 3.12 | 1.000 |

^a^$B$, coefficient; $SE$, standard error; $OR$, odd ratio; $CI$, Confidence interval
